# Changes in social inequalities in disability-free life expectancy in Southern Europe: the case of the Basque Country

**DOI:** 10.1186/s12939-014-0074-6

**Published:** 2014-09-20

**Authors:** Unai Martín, Santiago Esnaola

**Affiliations:** Department of Sociology 2, University of the Basque Country UPV/EHU, Basque Country, Spain; Department of Health, Basque Government, Vitoria-Gasteiz, Spain

## Abstract

**Background:**

Health expectancy is a useful tool to monitor health inequalities. The evidence about the recent changes in social inequalities in healthy expectancy is relatively scarce and inconclusive, and most studies have focused on Anglo-Saxon and central or northern European countries. The objective of this study was to analyse the changes in socioeconomic inequalities in disability-free life expectancy in a Southern European population, the Basque Country, during the first decade of the 21^st^ century.

**Methods:**

This was an ecological cross-sectional study of temporal trends on the Basque population in 1999–2003 and 2004–2008. All-cause mortality rate, life expectancy, prevalence of disability and disability free-life expectancy were calculated for each period according to the deprivation level of the area of residence. The slope index of inequality and the relative index of inequality were calculated to summarize and compare the inequalities in the two periods.

**Results:**

Disability free-life expectancy decreased as area deprivation increased both in men and in women. The difference between the most extreme groups in 2004–2008 was 6.7 years in men and 3.7 in women. Between 1999–2003 and 2004–2008, socioeconomic inequalities in life expectancy decreased, and inequalities in disability-free expectancy increased in men and decreased in women.

**Conclusions:**

This study found important socioeconomic inequalities in health expectancy in the Basque Country. These inequalities increased in men and decreased in women in the first decade of the 21^st^ century, during which the Basque Country saw considerable economic growth.

## Background

In the last decades, life expectancy has increased significantly in the south of Europe, as in most high-income countries [1]. However, the increase in life length does not necessarily imply an improvement in health at the population level. In order to obtain a broader picture of population health, we need to know whether the increase in life expectancy has been accompanied by an increase in the years of life in good health [2] or whether there has been an increase in the years of life with disability [3]. In addition, we have to analyse whether there has been improvement in all social groups, or whether there are social inequalities in health changes at the population level. Previous studies have shown that inequalities in life expectancy are increasing in most European countries [4-6], as well as in other high-income countries [7,8].

Health expectancy indicators address both of the aforementioned questions, combining in a single indicator, a measure of mortality and health status. Indeed, in contrast with population health analysis based exclusively on life expectancy, evidence on changes in health expectancy is not conclusive, with mixed, even contradictory, results depending on the population, the period studied and the health indicators used [9-11]. Regarding social inequalities in health at the population level, health expectancy indicators have proven useful [12-14], generally describing greater inequalities than those identified using classical measures of mortality. However, the evidence on changes in social inequalities in health expectancy is relatively scarce and inconclusive. Some studies have found an increase in inequalities between extreme groups [15,16], whereas in others the pattern is not clear when socioeconomic groups are considered [17] or a decrease in inequalities is observed [18].

In the European context, Southern European populations have shown relatively small socioeconomic inequalities in mortality since the nineteen eighties [19]. However, this mortality-based perspective has not been complemented with analyses of changes in inequalities in health expectancies, most studies focussing on Anglo-Saxon and central or northern European countries [15-18,20]. An analysis of changes in social inequalities in health expectancy in Southern European populations would allow a better characterisation of health inequalities, and improve our understanding of the underlying explanatory factors. We therefore studied socioeconomic inequalities in disability-free life expectancy, and their changes over the first decade of the 21st century in a Southern European region, the Basque Country (Spain).

## Methods

We performed an ecological cross-sectional study of temporal trends, focusing on the Basque Country population in 1999–2003 and 2004–2008. For each time period, we calculated two mortality indicators (all-cause mortality rate and life expectancy at birth), prevalence of disability, and three composite indices of mortality and health status: disability-free life expectancy at birth, life expectancy with disability, and percentage of life spent free of disability.

Mortality data were obtained from the Basque Institute of Statistics (EUSTAT).

Out of the total number of deaths in the period (187,944), 116 (0.06%) were not geographically referenced and were excluded from the analysis. Data on health were obtained from the 2002 and 2007 Basque Health Interview Surveys (ESCAV), carried out by the Department of Health of the Government of the Basque Country. These surveys collect information on various health determinants as well as health status in a sample of the Basque Country population living in private households. The methodology and variables used were the same in both years, with sample sizes of 14,787 in 2002 and 13,555 in 2007. More details of this survey have been published previously [21].

To obtain the socioeconomic status, a deprivation index was calculated for each census tract using the data of the 2001 Population and Housing Census carried out by the National Statistics Institute of Spain, and the methodology proposed by Dominguez-Berjón et al. [22] (principal component analysis), based on the socioeconomic indicators available for each census tract. Five simple indicators were included in this index: a) unemployment; b) low educational attainment; c) low educational attainment in young people; d) manual workers and e) temporary workers. The deprivation index was categorized in quintiles (I meaning lowest deprivation; V meaning highest deprivation). The deprivation index was assigned to each death and each of the survey’s participants based on individual residence.

Disability was used to measure health status. The ESCAV records level of disability in terms of chronic activity limitation using the following question: “Do you feel that your ability to carry out normal activities is limited due to any deficiency or health problem compared to others of your same age and sex?”

For each time period and area deprivation category, crude and standardised mortality rates and the standardised prevalence of disability were calculated by the direct method using the 2002 Basque population as the reference. To assess the association between the deprivation index and the health variable, age-adjusted prevalence ratios were obtained using log-binomial regression models. Life expectancy and the corresponding confidence intervals were calculated with the abbreviated life table according to the method of Chiang [23].

The disability-free life expectancy was calculated using Sullivan’s method [24], and the confidence intervals following the method proposed by the European Health Monitoring Unit [25]. Life expectancy with disability was obtained by subtracting the years of life expected to be spent disability free from the total life expectancy.

Several different relative and absolute measures were calculated to summarise the inequalities and to analyse changes over time [26]: the difference between extreme values; the relative and absolute population attributable risk, the slope index of inequality (SII), and the relative index of inequality (RII) using linear regression models. In all cases we took the least deprived quintile as the reference. All the analyses were carried out independently for men and women. The statistical analysis was performed using the SAS 9.2 programme.

## Results

In the period 2004–2008, life expectancy decreased with increasing deprivation of the area of residence (Table 1). In men, life expectancy in the least deprived quintile was 79.2 years, 2.8 years longer than in the most deprived quintile. In women, those living in the least deprived quintile had a life expectancy of 85.5 years, which was 0.8 years more than those living in the most deprived areas. Between the periods 1999–2003 and 2004–2008, both in men and women, life expectancy increased at all levels of deprivation, but the increase was smallest in the most advantaged quintile; thus, the differences between the deprivation quintiles fell.

**Table 1:** **Crude and standardized mortality rates and life expectancy at birth by sex, deprivation quintile and period**

	**1999-2003**			**2004-2008**		
**Deprivation index (Quintiles)**	**Crude mortality rate**	Standardized mortality rate (CI 95%*)	Life expectancy (CI 95%*)	**Crude mortality rate**	Standardised mortality rate (CI 95%*)	Life expectancy (CI 95%*)
**Men**						
1 (Least deprived)	8.8	11.3 (11.1-11.6)	77.8 (77.6-78.1)	9.0	10.1 (9.90-10.3)	79.2 (79.0-79.4)
2	9.3	12.0 (11.8-12.2)	76.9 (76.6-77.1)	9.2	10.6 (10.4-10.8)	78.3 (78.1-78.6)
3	9.5	12.2 (11.9-12.4)	76.4 (76.2-76.7)	9.2	10.7 (10.5-10.9)	78.1 (77.8-78.3)
4	10.2	12.5 (12.3-12.8)	76.0 (75.7-76.3)	10.3	10.9 (10.7-11.1)	77.8 (77.6-78.1)
5 (Most deprived)	10.7	13.4 (13.1-13.6)	74.8 (74.5-75.0)	10.7	11.8 (11.5-12.0)	76.4 (76.1-76.6)
**Women**						
1 (Least deprived)	8.7	6.3 (6.2-6.4)	84.4 (84.2-84.6)	8.8	5.7 (5.6-5.8)	85.5 (85.3-85.7)
2	8.1	6.4 (6.3-6.5)	84.2 (83.9-84.4)	8.2	5.8 (5.6-5.9)	85.3 (85.1-85.5)
3	7.6	6.4 (6.3-6.6)	84.1 (83.8-84.3)	7.7	5.7 (5.5-5.8)	85.4 (85.2-85.6)
4	8.4	6.7 (6.6-6.9)	83.6 (83.3-83.8)	8.5	5.9 (5.8-6.0)	84.9 (84.7-85.1)
5 (Most deprived)	8.0	6.8 (6.7-7.0)	83.3 (83.0-83.5)	8.2	5.9 (5.8-6.1)	84.7 (84.5-85.0)

*CI: Confidence Intervals.

The rate of disability increased with area deprivation (Table 2). Between the periods 1999–2003 and 2004–2008, inequalities increased in men, since the decrease in disability was greater among those living in the least deprived areas. In women, the inequalities between extreme groups remained stable.

**Table 2:** **Age-standardized prevalence of disability (%) and prevalence ratios (PR) by deprivation quintile, sex and period**

	**2002**		**2007**	
**Deprivation index (Quintiles)**	**Disability**	**PR (CI 95%)**	**Disability**	**PR (CI 95%)**
**Men**				
1 (Least deprived)	7.3	Reference	5.9	Reference
2	6.5	0.9 (0.7-1.2)	6.7	1.1 (0.8-1.5)
3	8.2	1.1 (0.9-1.5)	7.9	1.3 (1.0-1.8)
4	7.7	1.1 (0.8-1.4)	10.2	1.7 (1.3-2.3)
5 (Most deprived)	11.6	1.6 (1.2-2.0)	11.4	2.0 (1.5-2.6)
**Women**				
1 (Least deprived)	5.7	Reference	5.3	Reference
2	6.2	1.1 (0.8-1.4)	7.6	1.4 (1.1-1.8)
3	7.9	1.2 (1.0-1.6)	8.4	1.5 (1.2-1.9)
4	7.5	1.3 (1.0-1.7)	6.9	1.3 (1.0-1.7)
5 (Most deprived)	9.4	1.6 (1.3-2.0)	9.4	1.6 (1.2-2.0)

Disability-free life expectancy was higher in women and decreased as area deprivation increased (Table 3). In the period 2004–2008, men of the least deprived quintile had a disability-free life expectancy 6.7 years longer than those of the most deprived quintile, while in women, the difference reached 3.7 years. Moreover, life expectancy with disability was higher with increasing deprivation. While men living in the least deprived quintile expected to live 5.3 years with disability, for those in the most deprived area the figure was 9.1 years. In women, life expectancy with disability also increased with increasing deprivation, and the differences between the extremes was 2.9 years. As a consequence of these differences, the percentage of life spent disability-free decreased with increasing deprivation. Both in men and women, the inequalities in disability-free life expectancy between the extreme groups were greater than those in life expectancy.

**Table 3:** **Disability-free life expectancy (DFLE), absolute differences in DFLE, life expectancy with disability (LED), and percentage of life spent disability free (%DFLE) by sex, level of deprivation and period, and absolute change in the disability-free life expectancy**

	**1999-2003**				**2004-2008**				**Abs. change**
**Deprivation index (Quintiles)**	**DFLE (CI 95%)**	**Abs. diff DFLE (CI 95%)**	**LED**	**% DFLE**	**DFLE (CI 95%)**	**Abs. diff DFLE (CI 95%)**	**LED**	**% DFLE**	**DFLE (CI 95%)**
**Men**									
1 (Least deprived)	71.6 (70.7-72.6)	Reference	6.2	92.0	73.9 (73.0-74.8)	Reference	5.3	93.3	2.3 (0.4-4.1)
2	71.8 (71.0-72.6)	−0.2 (−2.0-1.6)	5.1	93.4	72.6 (71.8-73.5)	1.3 (−0.5-3.0)	5.7	92.7	0.8 (−0.9-2.5)
3	70.0 (69.1-70.9)	1.6 (−0.2-3.5)	6.5	91.6	71.5 (70.5-72.5)	2.4 (0.5-4.2)	6.5	91.6	1.6 (−0.3-3.5)
4	69.7 (68.8-70.6)	1.9 (0.1-3.8)	6.3	91.7	69.6 (68.6-70.7)	4.3 (2.3-6.2)	8.2	89.5	−0.1 (−2.0-1.9)
5 (Most deprived)	66.3 (65.3-67.3)	5.3 (3.4-7.3)	8.5	88.7	67.2 (66.2-68.3)	6.7 (4.7-8.6)	9.1	88.0	0.9 (−1.1-3.0)
**Women**									
1 (Least deprived)	77.6 (76.7-78.6)	Reference	6.7	92.0	79.2 (78.4-80.1)	Reference	6.3	92.7	1.6 (−0.2-3.4)
2	77.3 (76.5-78.1)	0.4 (−1.4-2.1)	6.9	91.9	76.9 (75.8-77.7)	2.5 (0.7-4.3)	8.5	90.0	−0.5 (−2.3-1.2)
3	76.5 (75.4-77.6)	1.2 (−0.9-3.2)	7.6	91.0	76.0 (74.8-77.0)	3.4 (1.4-5.3)	9.5	88.9	−0.6 (−2.8-1.6)
4	74.9 (74.0-75.8)	2.8 (0.9-4.6)	8.7	89.6	77.5 (76.5-78.5)	1.8 (−0.1-3.6)	7.4	91.2	2.6 (0.7-4.5)
5 (Most deprived)	73.3 (72.3-74.3)	4.4 (2.4-6.3)	10.0	88.0	75.6 (74.3-76.9)	3.7 (1.5-5.8)	9.2	89.2	2.3 (0.0-4.6)

Between the periods 1999–2003 and 2004–2008, the disability-free life expectancy increased significantly in men of the least deprived quintile (2.3 years IC95% 0.4-4.1) . In contrast, disability-free life expectancy increased significantly in women of the two most deprived quintiles. Consequently, the inequalities increased in men in absolute and relative terms (RII 1999–2003:9.1 RII 2004–2008: 11.5), and decreased in women both in absolute and relative terms (RII 1999–2003:7.4 RII 2004–2008:4.3) (Table 4).

**Table 4:** **Summary measures of inequality in disability-free life expectancy: absolute and relative differences between extremes, absolute and relative population attributable risk (PAR), slope index of inequality (SII), and relative index of inequality (RII) by sex, deprivation quintile and period**

	**1999-2003**	**2004-2008**
**Men**		
Absolute differences	5.3	6.7
Relative differences between extremes	8.0	9.9
Absolute PAR	1.7	2.8
Relative PAR	2.5	3.9
SII	−6.4	−8.2
RII	9.1	11.5
Ratio quintile 1/quintile 5	1.1	1.1
**Women**		
Absolute differences	4.4	3.7
Relative differences between extremes	6.0	4.9
Absolute PAR	2.3	2.2
Relative PAR	3.0	2.9
SII	−5.6	−3.3
RII	7.4	4.3
Ratio quintile 1/quintile 5	1.1	1.1

## Discussion

This study analysed changes in socioeconomic inequalities in health over the first decade of the 21st century in a Southern European region, the Basque Country (Spain). We used disability-free life expectancy, allowing us to obtain a comprehensive overview of the changes in inequalities in mortality and health status. Our results indicate that although the inequalities in life expectancy have decreased in both sexes, changes in inequalities in disability-free life expectancy were different in men and women. In men, the greatest reduction in disability was among those living in the least deprived quintile, which produced a larger increase in disability-free life expectancy in this group, and consequently the inequalities in disability-free life expectancy widened. In women, however, inequalities in disability remained unchanged, and therefore, the disability-free life expectancy increased more among the most deprived groups, slightly narrowing existing socioeconomic inequalities.

Although the heterogeneity in the methods used for the calculation of health expectancy limits the comparability of our results [14], they are relatively consistent with the findings of most other studies in various respects [27,28]. First, as the deprivation increased, life expectancy decreased, as did disability-free life expectancy, while the years with disability increased [16,30]. Second, inequalities in health expectancy were greater than those found in life expectancy [11,16,30]. Finally, the differences between men and women in disability-free life expectancy were smaller than in overall life expectancy [12]. Regarding the comparison with other countries, the inequalities in health expectancy in the Basque Country seem to be smaller than those found in a study carried out in England using a similar methodology [16]. This is in line with research indicating that there are fewer social inequalities in mortality in Southern Europe [14].

The slight decrease in inequalities in life expectancy contrasts with the pattern observed in other studies [5,6], while our results are consistent with studies that indicate no changes or an increase in inequalities in health status [31-34]. The increase in inequalities in health expectancy in men is consistent with the results of most of the studies [15,20,29], while the slight decrease in inequalities in health expectancy in women had been found less frequently [18].

Our data differ from other findings since a different pattern in the changes in inequalities in disability as well as in disability-free life expectancy are seen for men and women. To the best of the authors´ knowledge, there are no studies which show such marked differences in the evolution of disability-free life expectancy inequalities in men and women, though a few describe differences in the evolution of health inequalities [15,17,31].

The reasons behind this sex-specific pattern could be related to the different determinants of disability in each sex: work-related accidents, traffic injuries and circulatory diseases caused the greatest impact on men´s disability, while osteomuscular diseases and some types of tumours were especially important among women [35]. The analysis of the differences in the socioeconomic distribution of these determinants in men and women could shed some light to better explain why inequalities increased in men and remained stable in women in this period.

This study has the limitations inherent to all cross-sectional and ecological studies [36] that use data related to mortality, health, and socioeconomic indicators derived from census records. First, as in other studies based on health expectancy indicators [25], the use of a health questionnaire in homes does not provide information on institutionalized individuals, and it may overestimate the disability-free life expectancy, especially in the elderly population. However, analysis we carried out on census data (not shown) suggested that this potential bias is small, and of similar magnitude across socioeconomic groups. Another limitation is related to the design effect [37] derived from the complex sampling of the ESCAV that could not be taken into account to estimate sampling errors. Our analysis of the 2002 ESCAV, for which the necessary information was available, showed that the underestimation of errors in the assessment of the inequalities in health expectancy was negligible [38]. In addition, Sullivan’s method for analysing changes in health inequalities is based on prevalence, which may produce biased results. However, there is evidence that in populations like the one considered in this study, in which there are no sharp changes in mortality rates and disability, this method produces reliable estimates of changes in health expectancy. In fact, it is the method most commonly used [39] and most widely recommended in the absence of longitudinal data [40,41]. Finally, the use of area-based socioeconomic indicators may underestimate the magnitude of inequalities calculated with individual socioeconomic information [42]. Nevertheless, the use of deprivation indices has advantages over other indicators such as the level of education. In particular, social groups defined by education can vary considerably in size and composition over time, which complicate the analysis and interpretation of changes in health expectancy [17]. Further, in the present study, various summary measures of health inequalities were calculated, making it possible to describe the changes both in absolute and relative terms.

The absence of a clear decrease in disability-free life expectancy inequalities described in this study occurred during a period of relative economic growth, increasing spending on social protection, and decreasing unemployment rate and income inequality [43]. Indeed, some authors have shown that the relationship between the changes in the structural determinants and health inequalities is complex [44,45]. According to our results, the improvement of the socioeconomic indicators and the increase of social inequalities in disability and disability-free life expectancy in men occurred simultaneously. The relatively better findings for the most advantaged men could be due to the fact that they probably benefit to a greater extent from improved health-related conditions that accompany income distributions policies than any other social group [46].

## Conclusions

This study found considerable socioeconomic inequalities in health in the population of the Basque Country. As deprivation increased, overall life expectancy and disability free life expectancy decreased, while the years lived with disability increased. These inequalities widened in men in the first decade of the 21st century, during which the Basque Country saw considerable economic growth. In women, we observed a downward trend and this was attributable to a reduction in inequalities in life expectancy together with inequalities in disability remaining unchanged. The start of the economic crisis in 2008, with a substantial impact in Southern European countries, suggests that there is an even greater need to continue monitoring social inequalities in health using also these summary measures of population health.
